# The complete chloroplast genome sequence of *Saussurea polylepis* (Asteraceae), a vulnerable endemic species of Korea

**DOI:** 10.1080/23802359.2017.1375881

**Published:** 2017-09-11

**Authors:** Seon A. Yun, Hee-Young Gil, Seung-Chul Kim

**Affiliations:** Department of Biological Sciences, Sungkyunkwan University, Suwon, Gyeonggi-do, Korea

**Keywords:** Chloroplast genome, *Saussurea polylepis*, Korean endemic, vulnerable species

## Abstract

The complete chloroplast genome sequence of *Saussurea polylepis*, one of vulnerable and endemic species of Korea, was determined. The genome size was 152,488 bp in length with 37.7% GC content. It included a pair of inverted repeats (IRa and IRb) of 25,191 bp, which were separated by small single copy (SSC: 18,689 bp) and large single copy (LSC: 83,417 bp) regions. The cp genome contained 113 genes, including 80 protein-coding genes, 29 tRNA genes, and four rRNA genes. Phylogenetic analysis of the combined 80 protein coding genes and four rRNA genes showed that *S. polylepis* was closely related to *S. chabyoungsanica.*

*Saussurea* (ca. 400 species) is one of the largest genera in the tribe Cardueae and is widely distributed throughout the Holarctic (Lipschitz 1979). The most comprehensive infrageneric classification system was proposed by Lipschitz (1979), recognizing six subgenera and 20 sections. In the most recent floristic treatment of Korea, approximately 34 species have been recognized (Im 2007). Of ca. 34 species, nearly 40% (13 species) are endemic to Korea, mostly occurring as small-sized populations in isolated regions of high elevation mountains and small islands. While most species occur in the backbone of Korean mountain ranges known as Baekdudaegan, *S. polylepis* is restricted to small clustered islands in southwestern part of the Korean peninsula. Since only a few individuals are found in a total of 10 locations, it is recognized as vulnerable species [VU B2ab (iii, iv)] in the Korean Red List (National Institute of Biological Resources 2014).

Here, we report the complete chloroplast genome of *S. polylepis*. The plant materials were sampled from Hongdo Island, Korea. The voucher specimen has been deposited at the Sungkyunkwan University Herbarium (SKK044232). Total DNA was extracted using the DNeasy Plant Mini Kit (Qiagen, Carlsbad, CA), following the instructions from the manufacturer. Genomic sequencing by an Illumina Miseq (Illumina Inc., San Diego, CA) platform was performed and chloroplast genome was assembled using SPAdes 2.425 and CLC Genomics Workbench v.5.5.1 (CLC Bio, Aarhus, Denmark). The chloroplast genome of *S. polylepis* was annotated using the DOGMA (Wyman et al. 2004) and CpGAVAS (Liu et al. 2012). The tRNA regions were confirmed using tRNAscan-SE with default setting (Schattner et al. 2005). Annotation of the start/stop codons of protein-coding genes was done using BLASTX, Geneious v.10.2.2. (Biomatters Ltd., Auckland, New Zealand), and then manually corrected for intron/exon boundaries. Maximum-likelihood analysis, including nine species from tribe Cynareae and 11 species representing seven tribes of the family Asteraceae, was conducted based on concatenated 84 chloroplast coding genes using IQ-TREE v.1.4.2 (Nguyen et al. 2015) with 1000 bootstrap (BS) replications.

The complete chloroplast genome of *S. polylepis* (MF695711) was 152,488 bp with 37.7% GC content and contained two inverted repeat regions (IRa and IRb) of 25,191 bp, separating a large single copy (LSC) region of 83,417 bp and a small single copy (SSC) region of 18,689 bp. The chloroplast genome contained 113 genes, including 80 protein-coding genes, 29 tRNA genes, and four rRNA genes. Nineteen genes, including seven tRNA genes and four rRNA genes, were duplicated in the IR regions. The structure, gene order, gene content, and GC content of the *S. polylepis* are similar to those of Cynareae chloroplast genomes (Curci et al. 2016; Lu et al. 2016; Cheon et al. 2017).

The phylogenetic analysis of major tribes of Asteraceae showed that tribe Cynareae, including three species of *Saussurea*, were monophyletic (100% BS) (Figure 1) and *S. polylepis* was most closely related to congeneric species *S. chabyoungsainca* in Korea. A further detailed work is necessary to elucidate phylogenetic relationships among major lineages of highly complex and specious genus *Saussurea*.

**Figure 1.**
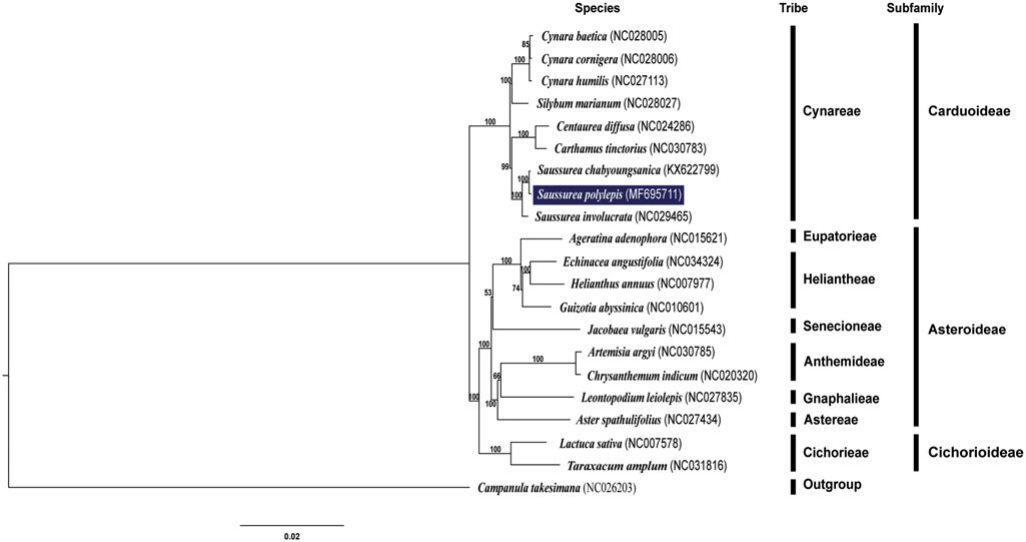
Maximum-likelihood tree based on 80 protein-coding and four rRNA genes from 20 representative species of Asteraceae. *Campanula takesimana* (Campanulaceae) was used as an outgroup and the bootstrap support values >50% are shown at the branches.
